# Minimally invasive surgical treatment of recurrent endometrial carcinoma: A systematic review

**DOI:** 10.1002/ijgo.70487

**Published:** 2025-08-21

**Authors:** Antonio Raffone, Daniele Neola, Alessio Colalillo, Claudia Tucci, Diego Raimondo, Antonio Travaglino, Maria Giovanna Vastarella, Massimiliano Fambrini, Maurizio Guida, Silvia D'Ippolito, Renato Seracchioli, Luigi Cobellis, Francesco Cosentino

**Affiliations:** ^1^ Department of Woman, Child, and General and Specialized Surgery University of Campania “Luigi Vanvitelli” Naples Italy; ^2^ Department of Neuroscience, Reproductive Sciences and Dentistry, School of Medicine University of Naples Federico II Naples Italy; ^3^ Università Cattolica del Sacro Cuore Rome Italy; ^4^ Division of Obstetrics and Gynecology, Department of Biomedical, Experimental and Clinical Sciences University of Florence Florence Italy; ^5^ Division of Gynaecology and Human Reproduction Physiopathology IRCCS Azienda Ospedaliero‐Universitaria di Bologna Bologna Italy; ^6^ Unit of Pathology, Department of Medicine and Technological Innovation University of Insubria Varese Italy; ^7^ Department of Medicine and Health Science “V. Tiberio” University of Molise Campobasso Italy; ^8^ Gynecologic Oncology and Surgery Unit Responsible Research Hospital Campobasso Italy

**Keywords:** endometrial carcinoma, laparoscopy, recurrence, robotic surgery

## Abstract

**Background:**

While the role minimally invasive surgery (MIS) is established for primary endometrial carcinoma (EC), its feasibility in recurrent cases remains underexplored.

**Objective:**

To systematically review the literature about MIS for EC recurrence.

**Search Strategy:**

A systematic literature search was conducted across six electronic databases, targeting studies published until October 31, 2024.

**Selection Criteria:**

Inclusion criteria encompassed all peer‐reviewed studies reporting MIS for recurrent EC.

**Data Collection and Analysis:**

Data extraction focused on surgical outcomes and survival metrics, following PRISMA guidelines.

**Main Results:**

Out of 9652 results, 15 studies with 17 cases of patients with EC recurrence met the inclusion criteria. All patients underwent successful MIS, with no intraoperative complications reported. Complete resection (when reported) was achieved in 100% of cases, and adjuvant treatment was administered in 64.7% of patients. The mean follow‐up duration was 23.6 months, with a disease‐free survival rate of 63.6%. Risk of bias assessment indicated a predominance of low to medium risk of bias within studies.

**Conclusion:**

MIS might be feasible and safe in cases of abdominal recurrence of EC when the number of recurrence localizations is less than three. MIS might be a management option independently from EC histology, grade and stage (except for stage IV), previous adjuvant therapy and group of risk. The endoscopic approach could be both laparoscopic and robotic, without any apparent difference in terms of feasibility, safety and survival outcomes. However, data on this topic are limited and our findings need to be confirmed by additional studies.

## 1 INTRODUCTION

Endometrial carcinoma (EC) is the fourth most common tumor in women worldwide. In 2024, approximately 67 880 new cases of EC were diagnosed, and 13 250 women died of this disease. The incidence of EC has exhibited a steady increase of about 1% yearly since the mid‐2000s indeed, likely attributed to an extended life expectancy and the increasing incidence of obesity in developed countries. Moreover, EC stands as the sole malignancy demonstrating a decrement in survival rates over the past four decades, with mortality rates continuing to rise at approximately 2% annually.^1^, ^2^ Although most EC cases are diagnosed at early disease stages and boast an encouraging overall five‐year survival rate of 81%, recurrences can occur within three years post‐primary treatment and dramatically affect survival.^3^

Management of EC recurrence involves a multidisciplinary approach with surgery, radiotherapy, and/or systemic therapy. It may vary depending on the site of recurrence, duration of disease‐free interval, patient's fitness and prior administration of adjuvant treatment such as radiotherapy, chemotherapy or both.^4^ Patients with local recurrence and no previous radiotherapy often receive radiotherapy, whereas women with disseminated distant metastasis receive multimodality treatment.^5^, ^6^ However, there is no agreement on the type of care options in patients with EC recurrence, probably due to the lack of robust evidence. In detail, the lack of data may be partially explained by the different patterns of recurrence (localized to the vagina, confined to the pelvis, or metastatic and involving the abdominal cavity or extra‐abdominal regions) and the heterogeneous nature of patients. Only one systematic review defines that the therapy of EC recurrence depends on the site of the recurrence, with surgery often favored, particularly for central pelvic, abdominal, and extra‐abdominal recurrences.^7^

The European Society of Gynecological Oncology, European Society for Radiotherapy and Oncology, and European Society of Pathology (ESGO‐ESTRO‐ESP) guidelines on EC management suggest that patients with recurrent disease should be considered for surgery only if complete resection of macroscopic disease can be achieved with a reasonable morbidity.^4^ Moreover, in women with EC recurrence eligible for surgery, another question regards the best surgical approach. In particular, although previous studies clearly demonstrate the superiority of a minimally invasive approach in primary surgery of EC, evidence concerning its feasibility in treating recurrences remains scarce^8^, ^9^, ^10^

The aim of the present study was to systematically review the literature about minimally invasive surgery (MIS) for EC recurrence.

## 2 MATERIALS AND METHODS

### 2.1 Study protocol and reporting

This study was designed as a systematic review of literature. A protocol was a priori built for systematically reviewing the literature. The review protocol was registered in the PROSPERO International Prospective Register of Systematic Reviews in October 2024 (registration no.: CRD42024602403).

All review stages, including search strategy, study selection, risk of bias assessment, data extraction and data analysis, were independently performed by two authors (AC and CT). In case of any disagreement, consensus was achieved by discussion among authors.

The whole study was reported following the Preferred Reporting Item for Systematic Reviews and Meta‐analyses (PRISMA) guidelines.^11^

Given the study design (i.e., systematic review of literature), IRB approval was not necessary for our Institution (Canadian Task Force Classification of Study Design Level III).

### 2.2 Search strategy and study selection

The start date of the literature search was November 1, 2024. Using several combinations of the topic‐related words (i.e., “metastas*”; “recurren*”; “endometr*”; “cancer”; “tumor”; carcinoma’; “neoplas”; “malignancy”; “laparoscop”; “minimally invasive”), six electronic databases (i.e., Google Scholar, Web of Sciences, Scopus, MEDLINE, ClinicalTrial.gov, and EMBASE) were searched from their inception to October 31, 2024 for all peer‐reviewed studies. To ensure literature saturation, we scanned the reference lists of included studies or relevant reviews identified through the search. The search string is reported in Supplementary material S1.

The selection of the abstracts was based on all the following criteria: being published in a peer‐reviewed journal, having accessible full‐text and being pertinent with the objective.

After the first abstract selection, two investigators (CT and AC) independently screened the full text. Corresponding authors were asked for additional information in cases where data provided in the published articles were insufficient.

### 2.3 Study selection criteria

We selected all randomized controlled trials (RCTs), including cluster RCTs, non‐randomized clinical trials (NRCTs), prospective and retrospective comparative cohort studies, case–control or nested case–control studies, cross‐sectional studies, case series and case reports that reported MIS for any EC relapse. In particular, we considered MIS as both laparoscopic and robotic‐assisted laparoscopic surgery.

The decision to include case reports, carrying a possible higher risk of bias, was taken because the rarity of the condition, the variability in recurrence patterns and the heterogeneous nature of the patient cohorts, may have determined a lack of multicenter prospective trials and so of available data.

Papers were excluded if of one of these criteria was identified: treatment of non‐recurrent EC; treatment of port‐site, lymph node or extra‐abdominal EC metastases; languages different from English.

### 2.4 Data extraction

Original data were extracted from the included studies without modifications. In particular, we extracted data about characteristics of the included studies (i.e., setting, study design, study period, sample size), patients (i.e., age, body mass index), treatment (i.e., surgical approach, surgical treatment and staging, uterus and adnexa route of removal, adjuvant therapy), primary tumor (i.e., histotype, The International Federation of Gynecology and Obstetrics [FIGO] grade, 2009 FIGO stage), recurrence site, adjuvant therapy type after surgical treatment of recurrence, surgical outcomes (i.e., perioperative complication and resection margins), oncological outcomes and follow‐up time.

### 2.5 Risk of bias within studies assessment

The risk of bias within studies was assessed adopting the methodological index for non‐randomized studies (MINORS) for NRCTs^12^ and the methodological quality and synthesis of case series and case reports.^13^

In particular, when the MINORS were used, seven applicable domains related to risk of bias were evaluated for each included study: (1) Aim (if the study had a clearly stated aim). (2) Inclusion of consecutive patients (if the patient selection included all eligible patients during the study period). (3) Prospective collection of data (if data collection was performed following a protocol a priori defined). (4) Endpoints appropriate to the aim (if authors reported data about localization of recurrence, MIS treatment, surgical and oncological outcomes and follow‐up). (5) Unbiased assessment of the study endpoint (if assessment of surgical, postoperative and follow‐up outcomes were unbiased reported). (6) Appropriate follow‐up period (if the follow‐up time was adequate). (7) Loss to follow‐up (if loss to follow‐up was less than 5%).

On the other hand, when the methodological quality and synthesis of case series and case reports was used, four applicable domains were evaluated: (1) Selection (does the patient[s] represent[s] the whole experience of the investigator [center] or is the selection method unclear to the extent that other patients with similar presentation may not have been reported?). (2) Ascertainment (was the outcome adequately ascertained?). (3) Causality (were other alternative causes that may explain the observation ruled out? Was follow‐up long enough for outcomes to occur?). (4) Reporting (is the case[s] described with sufficient details to allow other investigators to replicate the research or to allow practitioners make inferences related to their own practice?). Finally, an overall judgment based on the four domains was assessed (low, medium or high risk of bias).

## 3 RESULTS

### 3.1 Study selection

**FIGURE 1:**
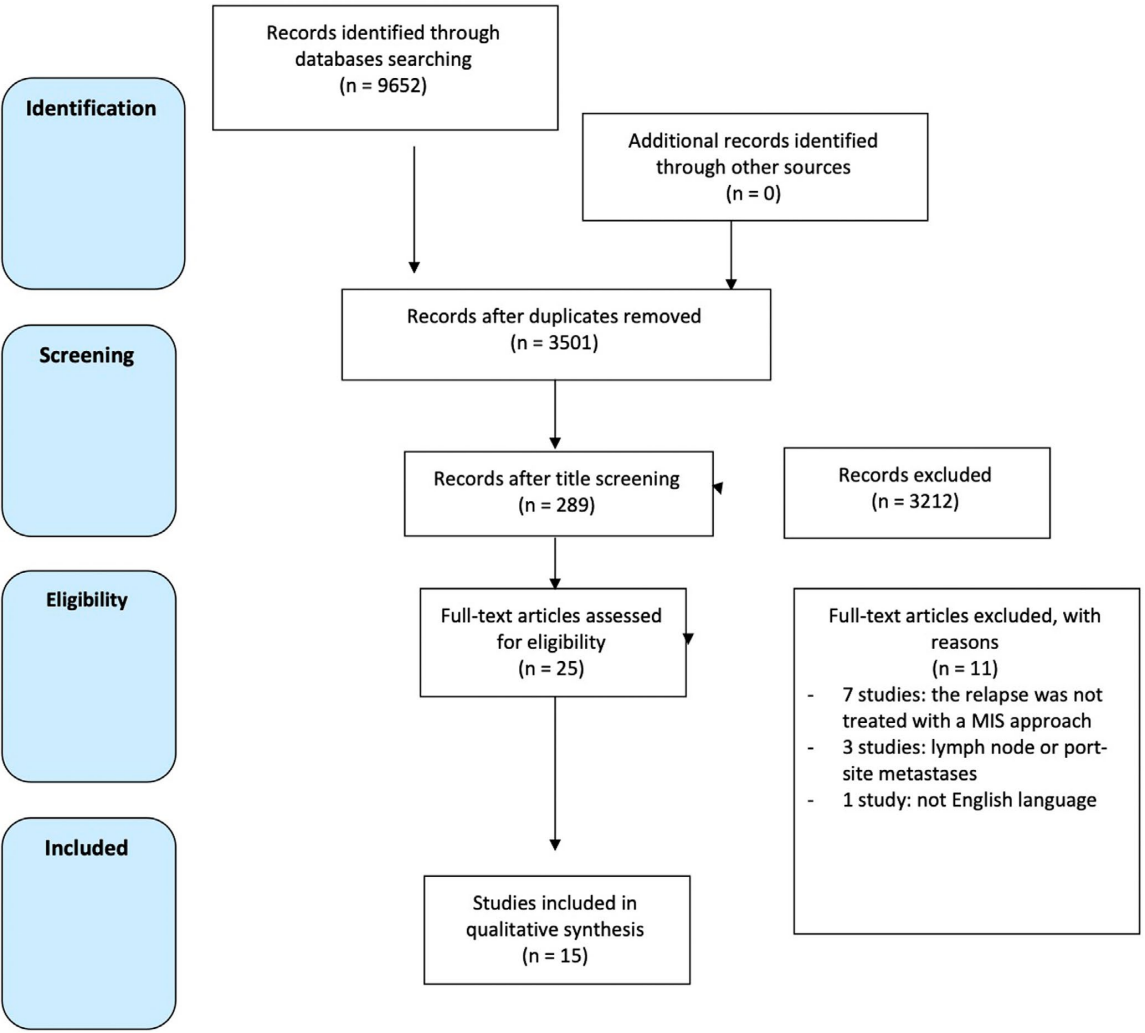
Flow chart of study selection step of the systematic review and meta‐analysis (Preferred Reporting Item for Systematic Reviews and Meta‐analyses [PRISMA]). Adapted from Moher et al.^34^

At the end of databases search, the research strategy retrieved 9652 results. Duplicate removal led to 3501 articles. Among them, the screening process yielded 25 selected papers to evaluate in full text. Based on the evaluation of the full text, we included 15 papers in the qualitative synthesis^14^, ^15^, ^16^, ^17^, ^18^, ^19^, ^20^, ^21^, ^22^, ^23^, ^24^, ^25^, ^26^, ^27^, ^28^ (Figure 1), with a total of 17 patients of MIS treated for EC recurrence.

### 3.2 Studies and patient characteristics

Of the total of the included studies, 12 studies were case reports,^14^, ^15^, ^17^, ^18^, ^19^, ^20^, ^21^, ^22^, ^23^, ^24^, ^26^, ^27^ of which three were video‐articles,^17^, ^18^, ^24^ one study was an observational prospective cohort study^25^ and two studies were observational retrospective cohort studies,^16^, ^28^ covering a time range from 2010 to 2024. The characteristics of the included studies and patients are reported in Table 1.

**TABLE 1:** Characteristics of included studies and patients.

Study	Study design	Age	Number of included patients/total of patients	BMI	Histotype of primary tumor	FIGO stage	FIGO grade	LVSI	ESGO‐ESTRO‐ESP risk group	Primary surgical treatment	Surgical approach of primary surgery	Adjuvant therapy
Izaki et al. (2009)^20^	Case report	55	1	‐	‐	IIIC	1	‐	High	TAH‐BSO + pelvic LND	Laparotomy	Chemotherapy (carboplatin and paclitaxel)
Choi et al. (2011)^15^	Case report	62	1	‐	Mucinous and squamous	IIIC	1–2	‐	High	TLH‐BSO + pelvic and paraaortic LND	Laparoscopy	Chemotherapy (cisplatinum)
Di Donato et al. (2011)^21^	Case report	70	1	71	Endometrioid	IB	2	‐	Intermediate	TAH‐BSO	Laparotomy	Brachytherapy
Rekhi (2015)^22^	Case report	39	1	‐	‐	II	3	‐	Intermediate/high	TAH‐BSO + pelvic LND + omentectomy	Laparotomy	Chemotherapy and radiation therapy
Gallotta et al. (2016)^17^	Case report (video article)	58	1	‐	‐	IB	2	‐	Intermediate	‐	‐	‐
Menderes et al. (2016)^18^	Case report (video article)	55	1	‐	Endometrioid	IIIA	‐	‐	High	Debulking surgery	Laparotomy	Chemotherapy (carboplatinum and paclitaxel) and brachytherapy
Gallotta et al. (2018)^24^	Case report (video article)	55	1	‐	Endometrioid	IB	2	‐	Intermediate	TAH‐BSO + pelvic LND + peritoneal washing	Laparotomy	‐
Mascilini et al. (2018)^25^	Prospective cohort study	55	1/51	‐	‐	‐	‐	‐	‐	‐	‐	Not specified
Da Dalt et al. (2019)^14^	Case report and narrative review	53	1	‐	Endometrioid	IIIA	2	‐	High	TAH‐BSO + pelvic LND	Laparotomy	Chemotherapy (carboplatin and paclitaxel)
Sozzi et al. (2020)^16^	Retrospective study	53 (median)	2/18	24 (median)	‐	‐	‐	‐	‐	‐	‐	Chemotherapy and radiation therapy Chemotherapy
Khadraoui et al. (2021)^23^	Case report	77	1		Endometrioid	IB	‐	‐	Intermediate/high	‐	‐	‐
Sano et al. (2021)^27^	Case report	45	1		‐	IA	1	Absent	Low	TLH‐BSO + pelvic LND	Laparoscopy	Observation alone
Di Donna et al. (2022)^26^	Case report	59	1	‐	Endometrioid	IA	1	‐	Low	TAH‐BSO	Laparotomy	Observation alone
Welp & Duska (2024)^19^	Case report	62	1	‐	‐	IA	1–2	Absent	Low	TAH‐BSO + pelvic LND + omentectomy + peritoneal washing	Laparotomy	Observation alone
Certelli et al. (2024)^28^	Retrospective study	63 76	2/20	‐ ‐	‐ ‐	‐ ‐	‐ ‐	‐ ‐	‐ ‐	‐ ‐	‐ ‐	‐ ‐
Total (%)	‐	39‐77	17	24‐71	7 Endometrioid 1 Mucinous	IA‐IIIC	1‐2 (90%)	Absent	Low (25) Intermediate (25) Intermediate/High (16.7) High (33.3)	TAH‐BSO + LND (50) TLH‐BSO + LND (20) TAH‐BSO (20) Debulking surgery (10) Omentectomy (20) Peritoneal washing (20)	Laparotomy (80) Laparoscopy (20)	Observation alone (27.3) Brachytherapy (9) Chemotherapy (36.4) Chemotherapy + radiation therapy (27.3)

*Note*: BMI, calculated as weight in kilograms divided by the square of height in meters.

Abbreviations: ‐, not reported; BMI, body mass index; ESGO, European Society of Gynecological Oncology; ESP, European Society of Pathology; ESTRO, European Society for Radiotherapy and Oncology; FIGO, The International Federation of Gynecology and Obstetrics; LND, lymph node dissection; LVSI, lymphovascular space invasion; TAH‐BSO, total abdominal hysterectomy and bilateral salpingo‐oophorectomy; TLH‐BSO, total laparoscopic hysterectomy and bilateral salpingo‐oophorectomy.

Regarding the included cases of EC recurrence, histotype was reported in seven (47%) studies: six (85.7%) were endometrioid, one (14.3%) was an adenocarcinoma with mucinous and squamous differentiation.

Tumor grading was reported in 10 (58.8%) studies: nine (90%) patients had FIGO grade 1–2 and one (10%) had FIGO grade 3.

The 2009 FIGO stage was reported in 12 (70.6%) studies: seven (58.3%) patients had stage I, one (8.3%) had stage II, and four (33.3%) had stage III.

We were able to calculate the class of risk according to the 2020 ESGO‐ESTRO‐ESP guidelines for endometrial cancer management^4^ in 12 (70.6%) patients: three (25%) were at low risk, three (25%) at intermediate risk, two (16.7%) intermediate/high risk, and four (33.3%) high risk.

Patients' age at the recurrence ranged from 39 to 77 years, with an average age of 58.6 years.

Surgical treatment of primary tumor was reported in 10 (58.8%) patients: it consisted of total abdominal hysterectomy (TAH) + bilateral salpingo‐ophorectomy (BSO) in two (20%) patients; TAH + BSO + lymph node dissection (LND) in five (50%) patients; total laparoscopic hysterectomy (TLH) + BSO + LND in two (20%) patients; debulking surgery in one (10%) patient; omentectomy in one (10%) patient and peritoneal washing in two (20%) patients.

Adjuvant treatment after primary surgery was reported in 12 (70.6%) patients and consisted of chemotherapy in four (36.4%) patients, radiotherapy in one (9%) patient, brachytherapy in one (9%) patient, chemo‐radiotherapy adjuvant treatment in three (27.3%) patients and observation alone in three (27.3%) patients.

The characteristics of EC recurrence are reported in Table 2. The recurrence occurred after a mean of 43.1 ± 48.4 months from surgery and involved from one to three sites of recurrence. A total of 13 (76.5%) patients had a single localization recurrence, two (11.8%) patients had recurrence in two different localizations (i.e., vaginal cuff and small bowel^17^ and vaginal cuff and spleen^24^), another patient (5.9%) had recurrence in three different localizations (a mass involving the distal sigmoid colon/upper rectum and two bilateral distal peri‐ureteral masses^23^). In particular, according to the classification by Zanfagnin et al.^6^ the sites of recurrence were: isolated vaginal cuff localization in 0 (0%) patients, central pelvic localization in 10 (58.8%) patients, and distant extra pelvic localization in seven (41.2%) patients. The MIS approach was laparoscopic in nine (52.9%) patients and robotic in eight (47.1%) patients, with no operative complications when this information was reported (10/15 studies [66.7%]). Four (26.7%) studies explicitly reported information about margins of resection at pathologic examination of the specimen: in all cases reported the margins of resection were disease‐free. Details of surgery are reported in Table 2.

**TABLE 2:** Characteristics of endometrial carcinoma recurrence oncological and surgical outcomes.

Study	Recurrence time from surgery, months (mean ± SD)	Site of recurrence	Surgical approach of recurrence	Adjuvant therapy after surgical treatment of recurrence	Surgical outcomes (perioperative complication and resection margins)	Oncological outcomes	Follow‐up time, months (mean ± SD)
Izaki et al. (2009)^20^	14	Adrenal gland	Laparoscopic adrenalectomy	Chemotherapy (carboplatin and paclitaxel)	No perioperative complications ‐	Disease‐free	66
Choi et al. (2011)^15^	10	Adrenal gland	Laparoscopic left adrenalectomy	Chemotherapy (doxorubicin)	‐	Second recurrence to liver with a complete response with doxorubicin	35
Di Donato et al. (2011)^21^	12	Central pelvis	Laparoscopic vaginal excision	Observation alone	No perioperative complications ‐	Disease‐free	36
Rekhi (2015)^22^	‐	Adrenal gland	Robotic adrenalectomy	Chemotherapy (paclitaxel)	‐	‐	‐
Gallotta et al. (2016)^17^	13	Vaginal cuff recurrence Small bowel (isolated distant metastasis)	Laparoscopic small bowel resection with intracorporeal anastomosis and partial colpectomy	Chemotherapy	No perioperative complications Free resection margins	Disease‐free	16
Menderes et al. (2016)^18^	45	Right hemidiaphragm (nodule)	Exploratory laparoscopy and resection of the hepatic dome/hemidiaphragmatic tumor nodule	‐	No perioperative complications Free resection margins	‐	‐
Gallotta et al. (2018)^24^	20	Vaginal cuff recurrence Spleen (isolated intraparenchymal metastasis)	Robotic resection of vaginal cuff Robotic splenectomy	Chemotherapy (carboplatinum and doxorubicin, Caelix)	No perioperative complications ‐	Disease‐free	2
Mascilini et al. (2018)^25^	18	Central pelvis	Robotic nodule excision on vaginal stump	‐	‐	Second recurrence to spleen	16
Da Dalt et al. (2019)^14^	36	Adrenal gland	Laparoscopic adrenalectomy	Observation alone	No perioperative complications ‐	Disease‐free	12
Sozzi et al. (2020)^16^	‐	Perivisceral fat, internal iliac vascular compartment, rectum, ileo‐coccygeal and pubo‐coccygeal and coccygeal muscles	Laparoscopic laterally extended endopelvic resection	Chemotherapy	‐ Free resection margins	‐	36
	‐	Perivisceral fat, internal iliac vascular compartment, rectum, ileo‐coccygeal and pubo‐coccygeal and coccygeal muscles	Laparoscopic laterally extended endopelvic resection	Chemotherapy	‐ Free resection margins	‐	2
Khadraoui et al. (2021)^23^	36	Distal sigmoid colon/upper rectum and bilateral ureters (tumor mass)	Robotic low anterior resection and partial bladder resection	‐	No perioperative complications Free resection margins	‐	‐
Sano et al. (2021)^27^	26	Left ureter (tumor mass)	Laparoscopic tumor around the ureter resection and peritoneal washing	Chemotherapy (paclitaxel and docetaxel)	No perioperative complications ‐	Disease‐free	2
Di Donna et al. (2022)^26^	108	Right obturator fossa, ureter, obturator nerve, pelvic muscles, bones side wall and external iliac vein and internal iliac vascular compartment (isolated metastasis)	Robotic debulking, with en bloc resection of the external iliac vein, internal iliac compartment, obturator nerve, partial sacral plexus fibers, and partial pelvic muscles and periosteum pelvic bones	Chemotherapy	No perioperative complications ‐	‐	‐
Welp & Duska (2024)^19^	179	Rectosigmoid colon	Robotic low anterior resection and diverting loop ileostomy	‐	No perioperative complications ‐	Disease‐free	5
Certelli et al. (2024)^28^	‐	Perirectal nodule	Robotic perirectal nodule resection	‐	‐ ‐	DFS17 Second recurrence to spleen treated with surgery and chemotherapy	60 (death)
	‐	Central pelvis	Robotic anterior evisceration	‐	‐ ‐	DFS9 Further recurrence Rectum, omentum and PE LNs treated with surgery and chemotherapy	19 (death)
Total (%)	43.1 ± 48.4	Isolated vaginal cuff (0) Central pelvic (58.8) Distant extrapelvic (41.2)	Laparoscopy (52.9) Robotics (47.1)	Observation alone (18.2) Chemotherapy (81.8)	No perioperative complications (100) Free resection margins (100)	Disease‐free (63.6) Second recurrence (36.4)	23.6 ± 20.7

Abbreviations: ‐, not reported; LNs, lymph nodes; PE, physical examination; SD, standard deviation.

Adjuvant treatment after recurrence surgery was reported in 11 (64.7%) patients and consisted of chemotherapy in nine (81.8%) patients and observation alone in two (18.2%).

Follow‐up time was reported in 13 (76.5%) studies: the mean of months of follow‐up was 23.6 ± 20.7. Disease‐free survival was reported in 11 (64.7%) patients: seven (63.6%) patients had no recurrence at the term of follow‐up, while four (36.4%) patients experienced further relapse, which were localized at liver,^15^ at rectum, omentum and pelvic lymph nodes^28^ and at spleen in two patients.^25^, ^28^

### 3.3 Risk of bias within studies assessment

Given the study design, the risk of bias within studies was assessed by the methodological quality and synthesis of case series and case reports for 12 studies,^14^, ^15^, ^17^, ^18^, ^19^, ^20^, ^21^, ^22^, ^23^, ^24^, ^26^, ^27^ and with the MINORS for three studies.^16^, ^25^, ^28^

Regarding the 12 studies assessed adopting the methodological quality and synthesis of case series and case reports, seven (58.3%) studies^14^, ^15^, ^19^, ^20^, ^21^, ^24^, ^27^ were judged at low risk and five (41.7%) studies at medium risk.^17^, ^18^, ^22^, ^23^, ^26^ In particular, in the “selection” domain, nine (75%) studies^14^, ^15^, ^18^, ^19^, ^20^, ^21^, ^24^, ^26^, ^27^ reported the whole experience of the center, while this information was not available in the remaining three (25%) studies.^17^, ^22^, ^23^ In the “ascertainment” domain, all the studies clearly stated the outcome.^14^, ^15^, ^17^, ^18^, ^19^, ^20^, ^21^, ^22^, ^23^, ^24^, ^26^, ^27^ In the “causality” domain, 11 (91.7%) studies ruled out alternative causes that may explain the observation,^14^, ^15^, ^17^, ^18^, ^19^, ^20^, ^21^, ^22^, ^23^, ^26^, ^27^ while one (8.3%) study^24^ did not; on the other hand, seven (58.3%) studies had an adequately long follow‐up,^14^, ^15^, ^17^, ^19^, ^20^, ^21^, ^27^ while five (41.7%) studies^18^, ^22^, ^23^, ^24^, ^26^ did not report follow‐up information. In the “reporting” domain, three (25%) studies described the cases only partially,^17^, ^18^, ^26^ while the remaining nine (75%) studies described cases with sufficient details to allow other investigators to replicate the research (Table 3).

**TABLE 3:** Assessment of the risk of bias within the included studies according to the methodological quality of case reports and case series item.

Study	Domains for evaluating the methodological quality of case reports and case series					
	Selection	Ascertainment	Causality	Reporting	Overall judgment	
	Does the patient(s) represent(s) the whole experience of the investigator (center) or is the selection method unclear to the extent that other patients with similar presentation may not have been reported?	Was the outcome adequately ascertained?	Were other alternative causes that may explain the observation ruled out?	Was follow‐up long enough for outcomes to occur?	Is the case(s) described with sufficient details to allow other investigators to replicate the research or to allow practitioners make inferences related to their own practice?	Risk of bias
Izaki et al. (2009)^20^	Yes	Yes	Yes	Yes	Yes	Low risk
Choi et al. (2011)^15^	Yes	Yes	Yes	Yes	Yes	Low risk
Di Donato et al. (2011)^21^	Yes	Yes	Yes	Yes	Yes	Low risk
Rekhi (2015)^22^	Not available	Yes	Yes	Not available	Yes	Medium risk
Gallotta et al. (2016)^17^	Not available	Yes	Yes	Yes	No	Medium risk
Menderes et al. (2016)^18^	Yes	Yes	Yes	Not available	No	Medium risk
Gallotta et al. (2018)^24^	Yes	Yes	No	Not available	Yes	Low risk
Da Dalt et al. (2019)^14^	Yes	Yes	Yes	Yes	Yes	Low risk
Khadraoui et al. (2021)^23^	Not available	Yes	Yes	Not available	Yes	Medium risk
Sano et al. (2021)^27^	Yes	Yes	Yes	Yes	Yes	Low risk
Di Donna et al. (2022)^26^	Yes	Yes	Yes	Not available	No	Medium risk
Welp & Duska (2024)^19^	Yes	Yes	Yes	Yes	Yes	Low risk

**FIGURE 2:**
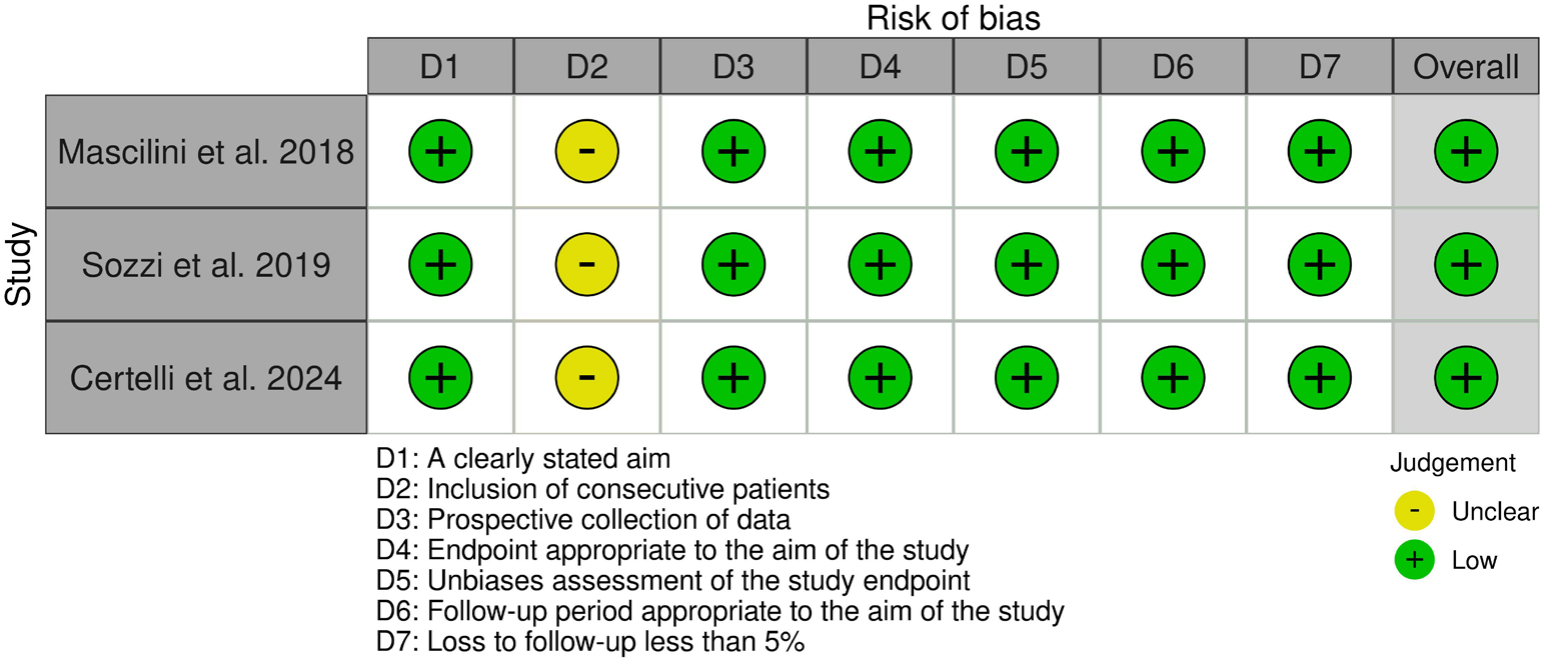
Assessment of the risk of bias within the included studies according to the methodological index for non‐randomized studies (MINORS).

Regarding the three studies^16^, ^25^, ^28^ assessed through the MINORS, all of them were categorized at low risk of bias for all domain, except the “patient selection” domain, since all three studies included not only recurrences of EC and it was unclear if all consecutive cases were considered (Figure 2).

## 4 DISCUSSION

### 4.1 Main findings

The present study shows that in cases of abdominal recurrence of EC, the MIS might be feasible when the number of recurrence localizations is less than three. In particular, it represents a viable option independently from histology, grade and stage (except for stage IV), previous adjuvant therapy and group of risk according to the 2020 ESGO‐ESTRO‐ESP guidelines.^4^ The endoscopic approach could be both laparoscopic and robotic, without any apparent difference in terms of feasibility, safety and survival outcomes.

### 4.2 Comparison with existing literature

The 2020 ESGO‐ESTRO‐ESP guidelines outline specific indications for surgical treatment in cases of recurrent EC.^4^ In particular, surgery can be considered in cases in which the recurrence is limited to the pelvis and can be fully resected, especially if the patient has not previously undergone radiation therapy.^6^ Pelvic exenteration might be indicated in carefully selected patients with a good performance status. For patients with isolated metastatic lesions (e.g., in the lungs, liver, or lymph nodes), surgical resection may be considered as part of a multidisciplinary approach, particularly if systemic disease control is achievable. Generally, surgery may be considered when recurrence is oligometastatic (recurrence with less than 5 metastases). Candidates for surgery should have an adequate performance status, controlled comorbidities, and minimal extra‐pelvic disease. Complete resection of the tumor is preferred if feasible, as it improves survival outcomes. Surgery is often combined with systemic therapy and/or radiation to optimize outcomes, depending on factors such as histology, previous treatments, and the patient's response. In recurrent cases in which surgery is feasible and aligns with the patient's overall treatment goals, a multidisciplinary team should evaluate the best approach to maximize survival and quality of life.^4^, ^29^ However, such guidelines do not express recommendation about surgical approach.

MIS, encompassing both laparoscopy and robotic techniques, might be a promising treatment option for recurrent endometrial carcinoma. The growing body of evidence suggests that this approach might be feasible as a safe alternative to traditional open surgery, especially for isolated recurrences in patients with manageable disease.^10^ The studies reviewed, although limited by small datasets, showed that MIS can be safely performed in cases where the recurrence is isolated and confined to technically approachable abdominal regions, with fewer than three metastatic sites.

Although Zanfagnin et al.^6^ recommended surgery only in cases of EC recurrence previously treated with adjuvant radiotherapy, our study found that only four surgically treated cases had undergone previous radiotherapy as adjuvant therapy. However, despite the majority of patients received surgical treatment independently from previous radiotherapy, favorable outcomes in terms of survival were achieved.

In terms of the localization of EC recurrences, the majority of cases in our study exhibited locoregional recurrence (central pelvic and vaginal cuff). Concerning extra‐pelvic recurrences, adrenal gland involvement was the prevailing site. Anyway, this prevalence did not consider isolated lymph node recurrence (i.e., location number 3 according to the classification by Zanfagnin et al.). In fact, MIS for isolated lymph node recurrence in gynecologic malignancies has already been reported as an option technically feasible, safe, and effective in terms of oncological outcomes, even for large tumors,^30^ and we opted to exclude this location of recurrence from our analysis.

In the literature it is well established that in the treatment of EC, robotic surgery outperforms laparotomy and is comparable to laparoscopy.^10^ Indeed, in the cases extracted in our review including only MIS treated cases, no perioperative complications were detected. Although we could hypothesize that this data could be overestimated by a publication bias in the literature, findings appear encouraging and worth investigating in future studies. Regarding obese patients, robotic primary surgery has shown superiority over laparoscopy in terms of hospitalization duration, postoperative complications, and quality of life.^31^ On these bases, we can hypothesize that robotic treatment for recurrence of EC in obese patients might outperform laparoscopic treatment. However, further studies will be required to substantiate this claim.

The most important independent prognostic factor for survival outcomes is the achievement of negative resection margins at surgical specimen pathologic examination.^8^ Accordingly, although in our systematic review only four of the included studies reported this data, all cases with free margins of resection had no further EC relapse.

Since the treatment of EC recurrence is multimodal, the role of chemotherapy after surgical treatment should also be further investigated. The standard chemotherapy is carboplatin and paclitaxel, while doxorubicin and paclitaxel are considered the most active therapies for second‐line chemotherapy.^4^ In our case series, the majority of patients received the standard of care chemotherapy. However, these findings might be revolutionized by the introduction of immunotherapy. In fact, in a phase 3, global, double‐blind, randomized, placebo‐controlled trial (RUBY trial), dostarlimab plus carboplatin‐paclitaxel significantly increased progression‐free survival among patients with primary advanced or recurrent EC, with a substantial benefit in the mismatch repair proteins deficient expression group of patients.^32^ Yet, in the phase 3 NRG GY018 study, the addition of pembrolizumab to standard chemotherapy has been recently proposed as first‐line treatment for patients with advanced stage or recurrent endometrial cancer regardless of mismatch repair status.^33^

Regarding survival outcomes, in the literature, the median progression‐free interval after secondary cytoreduction surgery of EC has been reported to be 50 months;^8^ however, in our systematic review, the median PFS appears to be relatively lower. This might be due to the short follow‐up in some included studies or to the study design in other included studies (i.e., case report). Anyway, the aim of our study was to assess feasibility and safety of MIS for EC recurrence, while impact of MIS on long‐term survival outcomes should be further investigated. In detail, the included studies collectively underscored the effectiveness of MIS in managing recurrent EC. Notably, many of them reported successful perioperative outcomes with no complications and clear resection margins in a subset of patients. The findings align with previous research demonstrating the superiority of MIS in primary EC treatment.

### 4.3 Strengths and limitations

While this may be the first systematic review that assesses the MIS for EC relapse highlighting the emerging trend of adopting minimally invasive techniques in the field, several limitations might affect the findings. First, our systematic review analyzed data from studies with different study designs, also including case reports. In particular, the reliance on case reports and small cohort studies raises concerns regarding the risk of publication bias and the generalizability of the findings, with possible overestimation of the results. Additionally, the absence of uniformity in reporting outcomes complicate data synthesis and interpretation. These limitations highlight the need for prospective data collection and registries on surgical approaches in recurrent EC, focusing particularly on the application of MIS in recurrent EC. However, the rarity of the condition, the variability in recurrence patterns and the heterogeneous nature of the patient cohorts make this task not easy in the future. In addition, future studies may compare surgical approach and immunotherapy as first‐line treatment for patients with recurrent EC.

## 5 CONCLUSION

MIS might be feasible and safe in cases of abdominal recurrence of EC when the number of recurrence localizations is less than three. MIS might be a management option independently from EC histology, grade and stage (except for stage IV), previous adjuvant therapy and group of risk. The endoscopic approach could be both laparoscopic and robotic, without any apparent difference in terms of feasibility, safety and survival outcomes.

However, given the limited literature available, our results should be read as preliminary and not generalizable. Additional studies are necessary to confirm these findings and to investigate the impact of MIS on long‐term survival outcomes in women with EC recurrence.

## AUTHOR CONTRIBUTIONS

AR, DN, AC and CT independently assessed electronic search, eligibility of the studies, inclusion criteria, risk of bias, data extraction and data analysis. AR, DN, AC, AT and MG contributed to the elaboration of methods for risk of bias assessment, data extraction and analysis. AR; AC, CT and FC conceived the study. MGV, MF and SDI worked on the design of the study. AR, DN, AC, CT and FC worked on the manuscript preparation. MG, SDI, RS, LC and FC supervised the whole study.

## FUNDING INFORMATION

None.

## CONFLICT OF INTEREST STATEMENT

The authors report no conflict of interest.

## Supporting information


Appendix S1:

Appendix S2:

Appendix S3:

## Data Availability

In accordance with the journal's guidelines, we will provide our data for independent analysis by a selected team by the editorial team for the purposes of additional data analysis or for the reproducibility of this study in other centers if such is requested.
