# Supplementary material for: Minimally invasive surgical treatment of recurrent endometrial carcinoma: A systematic review
Source: Int J Gynaecol Obstet. 2025 Aug 21;172(2):866–78. doi: 10.1002/ijgo.70487 (PMC13285899; doi:10.1002/ijgo.70487)
Supplement: Supplementary file 3 — Appendix S3: [file IJGO-172-866-s001.docx]

**Supplementary Material 1.** Full search string used for all databases.

(uter* OR endometr*) AND (cancer OR carcinoma OR neoplasm OR malignancy OR tumor OR tumour) AND (laparoscop* OR minimally invasive OR minimally-invasive) AND (recurrence OR relapse OR metastas*)
